# Understanding the Impact of Neural Variations and Random Connections on Inference

**DOI:** 10.3389/fncom.2021.612937

**Published:** 2021-06-07

**Authors:** Yuan Zeng, Zubayer Ibne Ferdous, Weixiang Zhang, Mufan Xu, Anlan Yu, Drew Patel, Valentin Post, Xiaochen Guo, Yevgeny Berdichevsky, Zhiyuan Yan

**Affiliations:** ^1^Electrical and Computer Engineering Department, Lehigh University, Bethlehem, PA, United States; ^2^Electrical and Computer Engineering Department, Beihang University, Beijing, China; ^3^Bioengineering Department, Lehigh University, Bethlehem, PA, United States

**Keywords:** bio-silicon computer, biological neural network, living neural network, spiking neural network, sparse connections, weight constraint, random network, recurrent network

## Abstract

Recent research suggests that *in vitro* neural networks created from dissociated neurons may be used for computing and performing machine learning tasks. To develop a better artificial intelligent system, a hybrid bio-silicon computer is worth exploring, but its performance is still inferior to that of a silicon-based computer. One reason may be that a living neural network has many intrinsic properties, such as random network connectivity, high network sparsity, and large neural and synaptic variability. These properties may lead to new design considerations, and existing algorithms need to be adjusted for living neural network implementation. This work investigates the impact of neural variations and random connections on inference with learning algorithms. A two-layer hybrid bio-silicon platform is constructed and a five-step design method is proposed for the fast development of living neural network algorithms. Neural variations and dynamics are verified by fitting model parameters with biological experimental results. Random connections are generated under different connection probabilities to vary network sparsity. A multi-layer perceptron algorithm is tested with biological constraints on the MNIST dataset. The results show that a reasonable inference accuracy can be achieved despite the presence of neural variations and random network connections. A new adaptive pre-processing technique is proposed to ensure good learning accuracy with different living neural network sparsity.

## Introduction

Artificial neural networks (ANN) have shown great success in solving real-world problems (Hinton et al., 2012; He et al., 2016; Silver et al., 2017). Most widely used neural network algorithms run on silicon- based computers, where the resource requirement and energy consumption become a challenge when the network size grows (Sze et al., 2017). In contrast, neurons and synapses naturally process information in a more energy-efficient way as compared to transistors and wires in computers (De Salvo, 2018). To develop a better artificial intelligent system, several groups (Reger et al., 2000; DeMarse et al., 2001; Han, 2013; Ju et al., 2015) proposed to incorporate biological living neural networks into the silicon platform to design a hybrid bio-silicon computer.

By dissociating the animal cortex into individual cells, placing them on an adhesive dish, and maintaining them in physiological conditions for several weeks, living neurons in a dish make random synaptic connections with each other and form an *in vitro* living neural network (Hasan and Berdichevsky, 2016). The *in vitro* neural cultures could respond to stimuli and be precisely controlled through microelectrode arrays (MEAs) (Thomas et al., 1972; Wang et al., 2006; Pizzi et al., 2007) or an optogenetics interface (Hong et al., 2015; Nguyen et al., 2019). Such a platform not only helps neuroscience studies but also makes it possible to use neurons as “devices” for learning and computing, which applies to image recognition (He et al., 2016), speech recognition (Hinton et al., 2012), and object detection (Zhao et al., 2019) tasks.

In an early hybrid bio-silicon design (Reger et al., 2000), neurons from the reticular formation of a lamprey brain were cultured and placed in a robot to guide its movement. The sensor data gathered from the robot were used as the input for the *in vitro* living neural network. Outputs of the living neural network were processed with silicon-based circuits and used to control the motor actuators of the robot. Experiments showed that, with the closed-loop interaction between the *in vitro* network and the robot, the robot’s behavior would adapt to the sensory information. Later works expanded the application sets for hybrid bio-silicon designs, Dranias et al. (2013) constructed a hybrid platform to process image patterns and Ju et al. (2015) showed that such a network is capable of classifying organized temporal sequences similar to music. Other works in this area were reviewed by Heard et al. (2018). Instead of using *in vitro* living neural networks that are randomly connected, researchers also tried to control *in vitro* neural connectivity and construct living neural circuits that carry out logic functions (Hasan and Berdichevsky, 2016). There were attempts to use *in vivo* neural networks for learning too (Musk and Neuralink, 2019). These works provided proof of concept that a hybrid bio-silicon network can perform some learning tasks and solve real-world problems.

However, the capability of the hybrid bio-silicon network is still far from the silicon-based design. Ju et al. (2015) constructed a comparison experiment between a hybrid bio-silicon design and a silicon-based design with a similar network structure and learning algorithm. The *in vitro* living neural network is modeled by a liquid state machine (LSM) structure (Maass et al., 2002) in the silicon-based design. For a temporal pattern classification task, the hybrid design achieved 60% classification accuracy, which is 10% lower than the LSM-based silicon design, and far below what a state-of-the-art silicon-based design (e.g., long short term memory; Hochreiter and Schmidhuber, 1997) could achieve. Although this result showed that a hybrid network can perform the learning task, the reason behind the accuracy gap has not been studied. In general, none of the well-designed benchmarks used to assess ANN performance have been tested in the bio-silicon platform due to implementation complexity.

The inferior performance observed in experiments could come from experimental limitations (e.g., control or recording precision), living neural network properties (e.g., high variations, random connections), as well as the poor learning capability of the algorithm. To achieve a better hybrid bio-silicon design, it is important to separate the influences of each factor and clearly understand the bottlenecks. While prior works mentioned above focused on implementation issues, this paper aims to study the impact of living neural network properties on prediction accuracy. Specifically, this work investigates the influence of neural variations and random connections on inference with an experimentally fitted biophysical model.

Contributions of the work are listed below: (1) This work proposes a new approach to the design of algorithms for living neural network implementation. Section “Living Neural Network Properties and Related Works” reviews related works and shows that none of the existing works had the same target as this paper, and none of the existing algorithms have been proved to be efficient for living neural networks. Since a living neural network has many unique properties that are not fully considered by previous works, rethinking the algorithm design with biological constraints is necessary. (2) A two-layer hybrid bio-silicon platform and a five-step design method are proposed for the living neural network algorithm study and introduced in section “Scope of the Study.” Characteristics of neurons in culture, including their variability, are captured in a biophysical model (section “Experiment Settings,” Experiment 1). The model is then transferred to a TensorFlow-based computational model through synapse weight and neuron threshold fitting to enable fast algorithm exploration (section “Experiment Settings,” Experiment 2). Accuracy between the biophysical and computational models are compared to validate that the model transfer does not lose fidelity (section “Experiment Settings,” Experiment 3). (3) A multi-layer perceptron algorithm (section “Algorithm”) is tested with biological constraints as a case study. The algorithm is adjusted for living neural network implementation with a new adaptive pre-processing technique (section “Experiment Settings,” Experiment 4), which helps the proposed neural network to achieve good learning accuracy for living neural networks with different sparsities. At last, neural variations are studied on the optimized model (section “Experiment Settings,” Experiment 5).

## Living Neural Network Properties and Related Works

Living neural networks have many intrinsic properties that are important for algorithm designs. Table 1 summarizes the living neural network properties and the difference between this work and prior bio-inspired algorithm designs. **Artificial neural network (ANN) algorithms**, which are based on the static numerical abstractions of the biological neural networks, have shown great potential on standard benchmark testing. Although the accuracy keeps improving for ANN designs, many important biological properties are omitted. For example, in living neural cultures, the information is coded, processed, and transferred through spikes. At a certain time, the output of a neuron can be a spike or no spike, depending on whether the membrane potential has crossed the threshold or not **(b1)**. However, in most ANNs, a neuron is modeled by an activation function such as a sigmoid or a rectified linear unit (ReLU), where the output could be a floating-point value. A recently proposed binary neural network (Courbariaux et al., 2016) did capture the threshold neuron output to make the algorithm more hardware efficient. However, in the binary neural network, detailed neuron dynamics such as neuron spike frequency adaptation and refractory period (Liu and Wang, 2001) **(b4)** are not modeled. In a living neural culture, the synaptic weights are positive or negative, depending on whether it is coming from an excitatory or inhibitory neuron, respectively (Chen and Dzakpasu, 2010) **(b2)**. The synaptic weights are also normally constrained to a range in living neural cultures, 2 × larger and 0.5 × smaller than the initial weights (Bi and Poo, 1998) **(b3)**. Both the neuron and the synapse have complicated dynamics and high variability **(b5)**. None of the existing ANN designs consider these. In a living neural culture, the network connections are randomly formed, the probability of connection between any pair of neurons is based on the distance between them **(b7)**. The overall connectivity of the network is typically less than 40% (Barral and Reyes, 2016) and a living neural network is very sparse **(b6)**. Recurrent connections also exist (Amit and Brunel, 1997) **(b8)**. Some network-level properties of a living neural network are captured by existing ANNs. For example, by utilizing recurrent connections, a network could “memorize” past content and be able to predict sequences. Another example could be pruning technology (Han et al., 2015), which cuts the unnecessary connections of a trained network to make the algorithm converge faster, as well as reduce the hardware cost. Overall, as summarized in Table 2, existing ANNs are designed for high performance and efficient hardware implementations **(a1)**. Hence, well-designed ANN algorithms may not be efficient when running on living neural networks because many necessary constraints are omitted.

**TABLE 1:** Living neural network properties and the comparison with other related works.

**Biological properties\works**		**MLP[1]**	**RNN [2]**	**Binary NN [3]**	**SNN [4]**	**RSNN [5]**	**LNN (Proposed)**
bl	Threshold neuron output				_√_	**_√_**	**_√_**
b2	Fixed synapse type				_√_	**_√_**	**_√_**
b3	Synapse strength constraint						**_√_**
b4	Neuron and synapse dynamics				**_√_**	**_√_**	_√_ (fitted)
b5	Neuron and synapse variability	_√_ [6]	_√_ [6]	_√_ [6]	**_√_**	**_√_**	_√_ (fitted)
b6	Sparse connectivity	_√_ [7]	_√_ [7]	_√_ [7]	_√_ 7	**_√_**	_√_
b7	Random connectivity					**_√_**	_√_
b8	Recurrent connectivity		_√_			**_√_**	_√_
Design goals		al	al	a2	a2	a2	a4

**The check mark C means one or more works in this category have captured the properties. [1]: LeCun et al. (1998), [2]: Hochreiter and Schmidhuber (1997), [3]: Courbariaux et al. (2016), [4]: Maass (1997), [5]: Maass et al. (2002), [6]: only synapse variation is modeled by randomly initialed weights, [7]: Han et al. (2015), [8]: Krizhevsky et al. (2012), [9]: Sacramento et al. (2018)*.*

**TABLE 2:** Different directions for bio-inspired algorithm designs*.

a1	Improve the learning capability for real word tasks, e.g., ANNs (MLP [1], LSTM [2], CNN [8])
a2	Improve hardware efficiency, e.g., binaryNN [3], pruning [7], SNN [4], RSNN [5]
a3	Provide hypothesis for biophysical mechanisms, e.g., dendritic backpropagation [9]
a4	Adjust or design algorithms for living neural network implementation, e.g., LNN (proposed)

*a1–a3 normally target on silicon implementation. Citations described in the caption of Table 1.

**Spiking neural network (SNN) algorithms** emulate spiking dynamics at different levels by using different neuron and synapse models. Because the spiking feature is captured, the network is “event-driven” rather than continuously processing. Therefore, SNNs are normally more energy efficient as compared to the ANN designs when running on hardware **(a2)**. However, the non-continuous threshold function for SNNs also brings challenges for the training algorithm design. The powerful backpropagation algorithm for ANNs needs to be adjusted for SNNs, and many prior SNN works focused on this direction (Lee et al., 2016; Huh and Sejnowski, 2018; Wu et al., 2019). Besides the backpropagation approach mentioned above, there are explorations on spike time-dependent plasticity (STDP)-based training algorithms and unsupervised learning approaches (Diehl and Cook, 2015; Iakymchuk et al., 2015). Another group of works tried to incorporate more biological properties into the computational models to provide new hypotheses for biophysical mechanisms **(a3)**. For example, Sacramento et al. (2018) capture more detailed neuron dynamics by modeling both the soma and dendritic compartments. This work provides a hypothesis that the dendritic micro-circuit provides a similar effect as the backpropagation algorithm in ANNs. Another example is the well-known blue brain project (Hill and Markram, 2008), which is trying to build biologically detailed digital reconstructions.

Unlike prior SNN works, where one or more properties are utilized for a unique design purpose, all living neural network properties need to be considered when targeting living neural network implementation **(a4)**. In this work, we call algorithms to be implemented on living neural networks **living neural network (LNN) algorithms**. This work focuses on understanding the impact of neural variations and random connections to LNN algorithm design, which is an important step toward building efficient bio-silicon computers.

## Algorithm Study Method

### Scope of the Study

**FIGURE 1:**
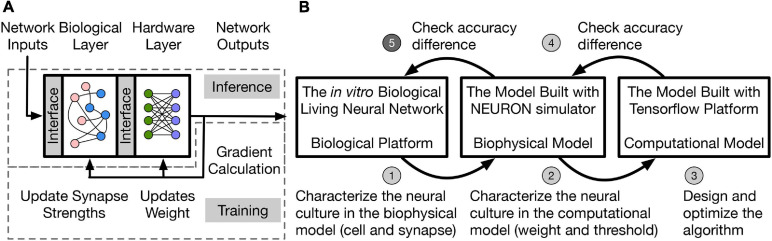
**(A)** Two-layer hybrid bio-silicon neural network. **(B)** A five-step method for LNN algorithm design. Step 5 (labeled with darker gray) of the method is out of the scope of the paper.

A two-layer hybrid bio-silicon neural network (Figure 1A) is targeted for algorithm study. The first layer is to be implemented in an *in vitro* living neural network and named **biological layer** in this paper. The second layer is computational and can run on general purpose computers or accelerators, and is named the **hardware layer** is this paper. More details about the network structure and design choice will be introduced in section “Network Structure and Data Representation.” To improve the algorithm exploration speed without losing fidelity, the *in vitro* biological living neural network (**biological platform** in Figure 1B is represented by two different models in this work. One is a **biophysical model**, which uses the NEURON simulator (Hines and Carnevale, 2001) to implement the biological layer. In this model, neurons are represented with the two-compartment Pinsky-Rinzel model (Pinsky and Rinzel, 1994), and synapses are represented with the alpha function model (Sterratt et al., 2011a). To further speed up the simulation, a **computational model** built with TensorFlow (Abadi et al., 2016) is used to model the living neural network, which uses the threshold function as the neuron activation function and models synapse as a floating-point value without dynamics.

In this work, the living neural network properties are converted into a simple computational model for fast algorithm design and optimization through a five-step method: (1) Biological experiments are conducted to determine neural and synaptic parameters; (2) the biophysical model captures the living neural network properties and variability by neuron and synapse parameter fitting from experimental data. However, the simulation speed for the biophysical layer is slow because of the large number of differential equations that are involved in modeling ion channels and synapses. Therefore, a simplified computational model with fast simulation speed is used and the living neural network properties are transferred into this model by fitting the weight and threshold distributions; (3) the learning algorithm is designed and optimized in the computational model with fitted biological layer parameters; (4)–(5) accuracy of the algorithm is checked on the biophysical model and the living neural network. Design details of steps (1)–(4) are introduced in the rest of the paper and step (5) remains as future work. This paper studies disinhibited networks and focuses on testing the influence of realistic biological properties on the inference process. Limitations and future steps of the work are discussed in section “Discussion.”

### Network Structure and Data Representation

A living neural network is randomly connected, which means other than the input-output connections, connections also exist between inputs, between outputs, and from outputs to inputs. As a result, the network has poly-synaptic (secondary) spikes triggered indirectly by the inputs, in addition to single-synaptic (primary) spikes. This paper assumes that an early “cut-off” mechanism for spike counting can be applied in experiments to distinguish the primary spikes from the secondary spikes, because the primary spikes normally happen before the secondary spikes. With this assumption, a living neural network can be observed as a feedforward network. In the biophysical and computational model, the biological layer is modeled with a 40% connectivity (Barral and Reyes, 2016). The hardware layer is fully connected. This work uses the MNIST dataset to evaluate network performance. Each MNIST image has 28 × 28 pixels in grayscale. Since controlling 784 input neurons is difficult, each MNIST image is compressed to 14 × 14 pixels. Section “Network and Algorithm Optimization Methods” describes the details of different pre-processing methods to compress the images. MNIST contains ten groups of digits, therefore, the network has 196 inputs, 10 outputs, and a varying number of hidden layer neurons.

**FIGURE 2:**
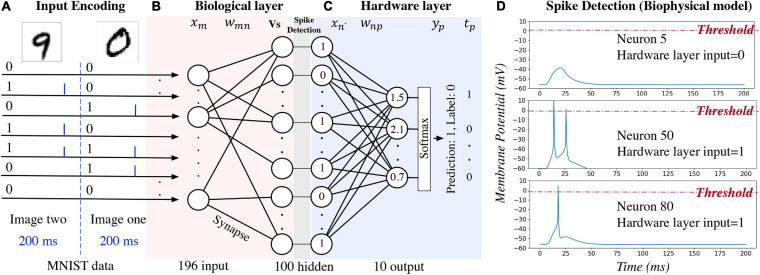
An example of using the hybrid neural network for digit recognition. The hidden layer is the output of the biological layer and the input of the hardware layer.

An example of using the hybrid neural network for digit recognition is shown in Figure 2. After pre- processing and compression, the pixel values are turned into binary (zero or one) and used as the network inputs to the biological layer (Figure 2A). Each input neuron corresponds to a pixel in the image. For the biophysical model, channelrhodopsin-2 (ChR2) (Nagel et al., 2003) is simulated as a light-gated ion channel. By controlling the light intensity and the duration, current (the blue bar in Figure 2A) is generated to evoke a single spike for the input neuron if the corresponding pixel value is one. Detailed settings used for our study are introduced in section “Experiment Settings,” Experiment 1. For one input image, currents are generated for different input neurons at the same time. This kind of deterministic binary data representation, instead of spike train representation, is used to distinguish the primary output spikes from the secondary output spikes. Data are represented as floating-point numbers for the hardware layer (Figure 2C) for both biophysical and computational model.

Network activity of the biological layer is measured by observing and converting the output neuron membrane potential to binary representations through a spike detection process in the biophysical model. In this model, membrane potential changes with time. A 200 ms window is set to observe the output spiking pattern for each input image. A detection example is shown in Figure 2D: membrane potential of neuron five does not reach the pre-defined threshold (0 mV); therefore, the output is zero and given as the input to the hardware layer. For neurons 50 and 80, the membrane potential exceeds the threshold, and the output value is one. For the computational model of the biological layer, the membrane potential is a floating-point value and it is converted to binary by comparing with a pre-defined threshold.

### Algorithm

**FIGURE 3:**
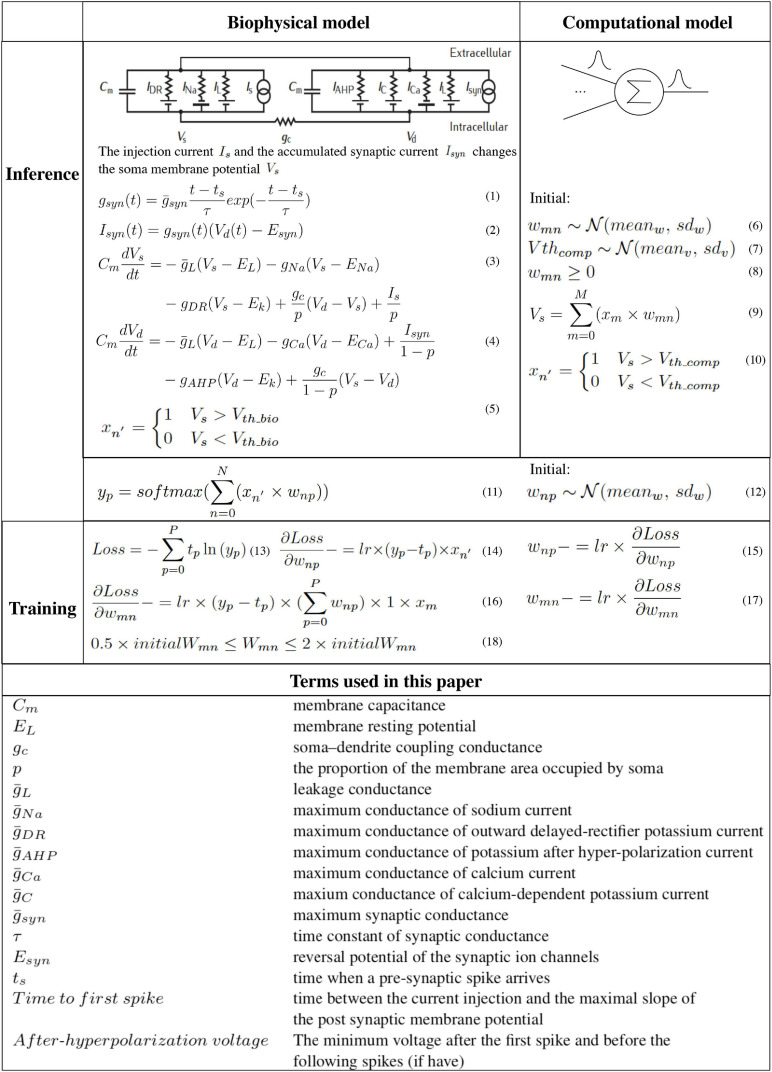
The hybrid network learning algorithm (Martin and Jurafsky, 2009; Sterratt et al., 2011a, b).

The algorithm study process using the biophysical and computational models is shown in Algorithm 1 with the pseudo-code. Corresponding equations are described in Figure 3. In order to faithfully model a living neural network, the biological layer uses realistic parameters derived from experimental characterization of *in vitro* neurons disassociated from the cortical region of a rat brain. The hardware layer provides both high-precision data representation and flexibility for weight updates, and hence has the potential to boost performance. This work only studies the disinhibited network behavior. Disinhibited environment is easily obtained by using GABA*_*A*_* receptor antagonists (Goel and Buonomano, 2013; Odawara et al., 2016) in *in vitro* experiments and it has been studied extensively in the literature. Both excitatory and inhibitory neurons exist in the network; however, synapses coming from the inhibitory neurons are prevented from functioning. Only excitatory synapses are captured for synapse parameter fitting. Correspondingly, we constrain the weights to be greater than zero in the biological layer in both biophysical and computational models to match the *in vitro* experiment setting.

**Algorithm 1:** Algorithm study process for biophysical and computational model.

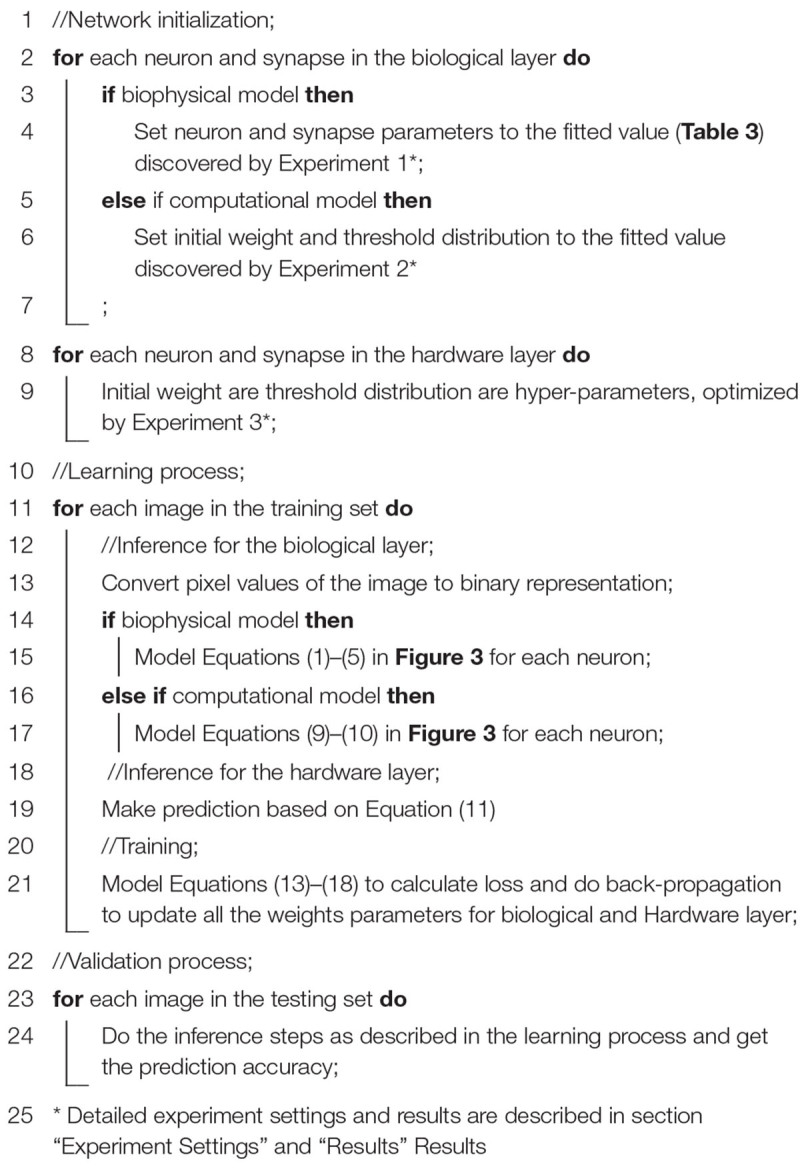

To capture the neuron dynamics in the biophysical model, a two-compartment Pinsky-Rinzel neuron model (Pinsky and Rinzel, 1994) with three somatic ion channels and four dendritic ion channels is used (Figure 3) (Equations 3 and 4). An alpha synapse model (Sterratt et al., 2011a) (Equation 1) is used. The two-compartment Pinsky-Rinzel model with the alpha synapse model reproduces a variety of realistic activity patterns in response to somatic current injection or dendritic synaptic input, which is verified by biological experiment results. Having more compartments will increase computation complexity, while a single- compartment model cannot capture some important neuron properties such as spike frequency adaptation behavior.

For pre-synaptic neurons, the current evoked by ChR2 (*I*_*s*_) is the input to the network. For post-synaptic neurons, an action potential is triggered when the accumulated synapse current (*I*_*syn*_) (Equation 2) is large enough. In the biophysical model, a spike occurs when the somatic voltage (*V*_*s*_) exceeds zero (Equation 5), which happens near the peak of the action potential. For the computational model, weights and the neuron thresholds for the biological layer are initialized following a normal distribution (Equations 6 and 7), and no negative weight is allowed (Equation 8). If the sum of input-weight products at a certain neuron exceeds the threshold (Equations 9 and 10), a spike is generated. The hardware layer is fully connected with ten outputs (Figure 2), the weights are initialed following a normal distribution without any constraint (Equation 12). A softmax function (Martin and Jurafsky, 2009) is used to normalize the output (Equation 11). The index of the largest output is the prediction result. The cross-entropy loss (Martin and Jurafsky, 2009) is used for error backpropagation (Equation 13). The gradient of the non-differentiable hard threshold function is estimated as a constant one, which is known as the “straight-through estimator” (Bengio et al., 2013) (Equations 14–17). The weights of the biological layer are restricted to the range of 0.5 *×* –2 × of initial weights (Equation 18).

### Experiment Settings

#### Experiment 1: Biophysical Model Parameter Fitting

To capture the living neural network variations in the biophysical model, parameters of the neuron and synapse models are fitted to data obtained through intracellular recordings. Nine different neurons and 12 different excitatory synapses are captured. Neural cultures were obtained by dissociating cortices of postnatal day 0 Sprague Dawley rats and plating neurons onto poly-D-lysine coated tissue culture dishes. On days *in vitro* (DIV) 12–19, neuron IV characteristics were obtained by injecting currents from −200 to 300 pA in current clamp mode with a 25 pA delta current step. Biophysical neural model (Pinsky-Rinzel) parameters were then adjusted for nine recorded cells through a multi-objective optimization approach. The defined mean square error incorporates different neuron features such as time to first spike, number of spikes, and after-hyperpolarization voltage produced by current injection. Excitatory postsynaptic currents (EPSCs) were evoked by patterned blue light stimulation of ChR2-expressing pre-synaptic neurons. The light power is set to 10*mW/mm*^2^ and the duration is 5 ms to evoke a single spike. Synaptic parameters were then extracted by fitting an alpha function to experimentally obtained EPSC waveforms for 12 different post-synaptic neurons and the average of 10 trails are reported.

#### Experiment 2: Computational Model Parameter Fitting

To reduce the computational complexity, this work further converts the biophysical model into a computational neural network model with a threshold activation function. To ensure that a similar accuracy can be achieved after the conversion, this paper uses the minimum number of pre-synaptic neurons that trigger a post-synaptic neuron to fire (minPreNum) as the bridge to convert the variations in the biophysical model to the variations in the computational model. The following experiment is conducted in the biophysical model: the number of pre-synaptic neurons is varied from 1 to 20 and the pre-synaptic neurons are stimulated through injected current (*I*_*s*_). The input neurons and synapses are randomly selected from the fitted excitatory neurons and synapses, respectively. The post-synaptic neuron is sequentially selected from all the fitted neurons. A selection is allowed to repeat. The experiment is repeated 1,000 times in simulation. The outcome of this step is nine minPreNum curves for each post-synaptic neuron. The threshold and weight variations are assumed to follow a normal distribution. The expectations of each of the minPreNum curves are used to estimate the threshold variation. The nine minPreNum are aligned with peak and averaged to estimate the weight distribution. The detailed calculation process and results are shown in section “Computational Model Parameter Fitting.”

#### Experiment 3: Accuracy Comparison Between Biophysical and Computational Models

To validate the computational model against the biophysical model, the handwritten digit recognition task is performed with both the computational and biophysical models. In this experiment, 100 MNIST images are used, and the network size is 196-100-10 for the input-hidden-output layer. As a first step, the computational and biophysical models are compared without any variations. In this experiment, a fitted neuron and a fitted synapse are chosen from Table 3. The weights in the computational model are initialized to the gsyn value of the fitted synapse without variation. *V th* in the computational model is set to match the behavior of the selected biophysical model. The network connectivity, topology, and the hardware layer weights are initialized to exactly the same for the biophysical and computational models. For the second step, both models with all variations are tested. The experiment goal is to check whether the network accuracy matches between biophysical and computational models with and without variation.

**TABLE 3:** Parameters fitting results.

	**Neuron parameters**									
Cell*	**1**	**2**	**3**	**4**	**5**	**6**	**7**	**8**	**9**	**Mean**
*c*_*rn*_ (μ*F*/*cm*^2^)	10	8	10	15	15	10	8	10	8	10.44
*E*_*L*_ (*mV*)	−45	−40	−45	−35	−45	−56	−50	−60	−60	−48.44
*ḡ_*L*_* × 10^–3^ (*S*/*cm*^2^)	1.10	0.85	1.48	1.15	1.48	2.10	0.8	1.10	0.70	1.20
*ḡ_*Na*_* × 10^–2^ (*S*/*cm*^2^)	8	6	15	29	25	9	7	11	8	13.11
*ḡ_*DR*_* × 10^–2^ (*S*/*cm*^2^)	2	2	0.5	2	1	9.9	2	12	6	4.16
*ḡ_*AHP*_* × 10^–2^ (*S*/*cm*^2^)	1	0.9	0.1	0.45	0.1	3	0.5	0.5	12	2.06
*ḡ_*Ca*_* × 10^–2^ (*S*/*cm*^2^)	0.8	0.8	0.8	0.8	0.8	0.8	0.8	0.8	0.8	0.80
*ḡ_*C*_* × 10^–2^ (*S*/*cm*^2^)	2	2	2	2.5	10	20	2	2	10	5.83

*Cell diameter is 20 um, p is 0.5, and g_*c*_ is 8 (S/cm^2^) for all cells.

	**Synapse parameters**												
**Cell**	**1**	**2**	**3**	**4**	**5**	**6**	**7**	**8**	**9**	**10**	**11**	**12**	**Mean**

*gsyn* (10^−3^μ S)	0.72	0.59	0.34	0.73	0.69	0.63	1.15	0.21	1.17	2.28	0.59	0.41	0.79
τ	4.50	5.70	4.55	7.80	5.75	6.96	5.95	6.00	4.40	4.75	4.90	6.99	5.69
Delay (ms)	1.80	2.00	2.00	2.00	2.00	1.30	2.00	2.00	1.40	1.30	2.00	2.00	1.82

#### Experiment 4: Network and Algorithm Optimization

The computational model is optimized to achieve good accuracy with fitted weight and threshold distributions for the biological layer. In these experiments, 1000 MNIST images are used, and there are 196, 100, and 10 neurons in the input, hidden, and output layers, respectively. Methods for network and algorithm optimization are proposed and described in section “Network and Algorithm Optimization Methods.” Experiment results are described in section “Network and Algorithm Optimization.”

#### Experiment 5: Neural, Synaptic, and Network Variation Study

After network and algorithm optimization, testing accuracy is validated on the full MNIST dataset and the influence of neuron variation, synapse variation, and weight constraint are studied. These experiments are based on 60,000 training samples and 10,000 testing samples. The network has 196 inputs and 10 outputs. Hidden layer neurons vary between 100 AND 2,000.

#### Experiment 6: Accuracy Comparison Between Biophysical and Computational Models After Optimization

The adaptive pre-processing approach developed through computational model optimization is then validated on the biophysical model. A hundred MNIST images are used for validation. The experiment settings are similar to those used in Experiment 3, except that the adaptive pre-processing optimization is applied with *Nin*_*b*_ = 20.

### Network and Algorithm Optimization Methods

The image classification accuracy of the fitted computational model (the result of Experiment 2) is lower than the state-of-the-art report on the full MNIST dataset. After a closer examination of the hybrid network, **one hypothesis is that** the average percentage of firing neurons in the hidden layer (*Nf*_*hidden*_) during the learning process directly influences the network capacity and hence the accuracy. When half of the hidden layer neurons spike on average, the network has the best learning capability. An intuitive example is that, if none of the neurons in the hidden layer spike, no matter what images are given from the dataset, the network will not learn at all. A similar situation also happens if all of the neurons in the hidden layer spike, regardless of the input image. For further analysis, considering the network structure in Figure 2, we introduce a parameter that is related to *Nf*_*hidden*_:

$$f=\left(S\,p\,a\,r\,s\,i\,t\,y\times N\,i\,n_{b}\times m\,e\,a\,n\,W\right)/V\,t\,h,\left(19\right)$$

where *Sparsity* is calculated by the number of connected neuron pairs divided by the total number of neuron pairs between the input- and the hidden-layer neurons, *Nin*_*b*_ represents the number of black pixels in the input images, *meanW* represents the average weights between input- and the hidden-layer neurons for the entire dataset after training, and *V th* is the average threshold of the hidden layer neurons. **The second hypothesis is that** when *f* = 1, the network has on average around 50% *×* Nhidden firing neurons for all of the images within the dataset. This is because Sparsity *×* Nin_b *×* meanW represents the expectation of xn in Equation (5), where $x_{n}=\sum_{m=0}^{M}x_{n}\times w_{m\,n}$. When Sparsity *×* Nin_b *×* meanW = Vth, a neuron in the hidden layer has on average 50% possibilities to fire. When *f* approaches zero, it is likely that none of the hidden layer neurons will fire, while when *f* is much larger than one, it is likely that all of the hidden layer neurons will fire. The optimization goal is to keep *f* close to one because the hybrid network has a greater learning capability when 50% of the hidden layer neurons are firing.

In this paper, an **adaptive pre-processing (Adpp) method** for the living neural network is proposed to shift *Nf*_*hidden*_ to 50% with a certain sparsity. In this approach, the input images are processed to achieve a target *Nin*_*b*_, so that the *f* value is close to one. We adopt a filter-and-pool approach (LeCun et al., 1998) as the pre-processing mechanism. It is sometimes necessary to compress the input data to fit the input-bio interface of the biological layer. Fortunately, the compression can be naturally incorporated into the pre- processing. The proposed work uses 196 neurons as the inputs. To compress the 28 *×* 28 MNIST images to 14 *×* 14, a specific filter size, stride, padding, and compression threshold need to be chosen. Relationship between these parameters are given by *compressed size* = $\frac{\left(o\,r\,i\,g\,i\,n\,a\,l\,s\,i\,z\,e-f\,i\,l\,t\,e\,r\,s\,i\,z\,e+2\times p\,a\,d\,d\,i\,n\,g\right)}{s\,t\,r\,i\,d\,e}+1$. The compression threshold that turns the greyscale value to black and white after the average pooling is tuned to meet the desired *Nin*_*b*_ value. By using the Adpp approach, *Nf*_*hidden*_ can usually be tuned to around 50% for a typical living neural network sparsity, and thus a good accuracy can be achieved. The *f* value is close to one when *Nf*_*hidden*_ is close to 50%.

Besides the adaptive pre-processing method, this work studies different **gradient estimator (Est) methods** to further improve the network accuracy. Because the hidden layer of the hybrid network uses a non-continuous threshold function, the gradient needs to be estimated. The straight-through estimator (Bengio et al., 2013), which considers the gradient as a constant one, is used for the previous experiments in this work. However, setting the gradient as one only when *x*_*n*_ (Figure 3) is within a small range can improve the training of the network. A set of gradient ranges around *V th* are explored in the experiment to find the best one. Finally, the biological layer learning rate and the hardware layer parameters such as the initial weights and learning rate can be tuned as hyper-parameters.

## Results

### Biophysical Model Parameter Fitting

Biophysical model fitting results for nine neurons and 12 synapses are shown in Table 3.

### Computational Model Parameter Fitting

**FIGURE 4:**
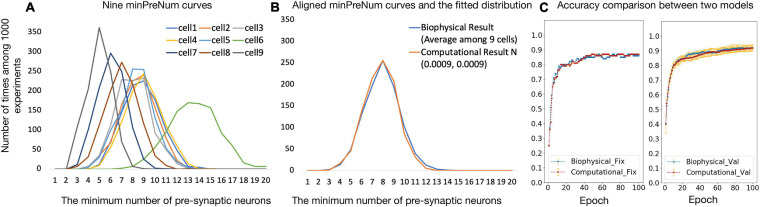
**(A,B)** Computational model parameter fitting; **(C)** accuracy comparison between biophysical and computational models.

Figure 4A shows the nine minPreNum histograms corresponding to each post-synaptic neuron. The average of the nine minPreNum expectations is 8.2, and the standard deviation is 2.4. These values are multiplied with the average maximum synapse conductance (the average gsy = 0.0008 in the supplementary materials is used to estimate the average weights) to derive the threshold distribution *N* (0.0066, 0.0019). To estimate the weight distribution, the nine minPreNum are aligned with the peak and the aligned points on the curves are averaged into one curve in Figure 4B. To fit the mean and standard deviation of the curve, the minPreNum experiment is repeated using the computational model. A post-synaptic neuron with threshold = 0.0066 is used. A weight distribution of *N* (0.0009, 0.0009) has the best fit.

### Accuracy Comparison Between Biophysical and Computational Model

To validate the computational model against the biophysical model, the digit recognition task is performed with both the computational and the biophysical models. Experiment settings are listed in Table 4 and the testing accuracy are compared in Figure 4C, which shows that the accuracy results for the biophysical and the computational models closely match each other with and without variations.

**TABLE 4:** Simulation parameters.

Experiment/dataset/network	**Accuracy comparison between two models**/100 MNIST images, training set same as testing set/size:196-100-10
Bio layer (fix)	Sparsity: 40%, *Vth_*cp*_: N* (0.0055, 0), *initW*_*cp*_: *N* (0.00072, 0), Lr: 1e-4
Bio layer (var)	Sparsity: 40%, *Vth_*cp*_: N* (0.0066, 0.0019), *initW_*cp*_: N* (0.0009, 0.0009), Lr: 1e-4
Hardware layer	Fully connected, *initW*: *N* (0.0009, 0.0009), Lr: 1e-2
Optimization (Experiment 6)	Adpp: *Nin*_*b*_ = 20
Experiment/dataset/network	**Network and algorithm optimization**/1,000 MNIST images, training set same as testing set/size:196-100-10
Bio layer	Sparsity: 40%, *Vth*_*cp*_: N (0.0066, 0.0019), *initW*: N (0.0009, 0.0009), Lr:5e-6
Hardware layer	Fully connected, *initW*: N (0.0009, 0.03), Lr: 0.008
Optimization	Adpp: *Nin*_*b*_ = 20, Estimator range: (0, 0.0075), Adlr: bio layer initial 5e-06, hardware layer initial 0.1, decay rate 0.1
Experiment/dataset/network	**Variation study**/60,000 and MNIST images for training and 10,000 for testing/size:196-(100–2,000)-10
Bio layer	Sparsity:40%, *Vth*_*cp*_: N (0.0066, 0.0019), *initW*: N (0.0009, 0.0009), Lr: 5e-6
Hardware layer	Fully connected, *initW*: N (0.0009, 0.03), Lr:0.008
Optimization	Adpp: *Nin*_*b*_ = 26, Estimator range: (0, 0.0075), Adlr: bio layer initial 1e-05, hardware layer initial 0.1, decay rate 0.1

### Network and Algorithm Optimization

**FIGURE 5:**
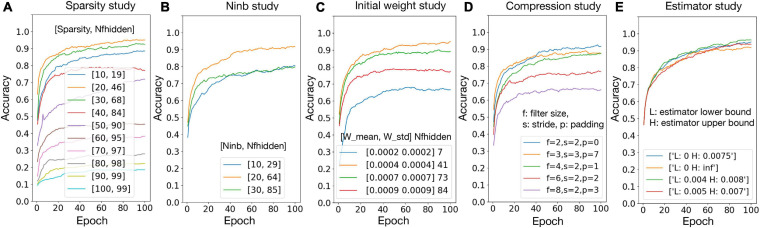
Network and algorithm optimization study. *Nf*_*hidden*_: average percentage of firing neurons in the hidden layer; *Nin*_*b*_: number of black pixels in the input images; W mean/W std: mean/standard deviation for biological layer initial weight distribution.

To validate the hypotheses proposed in section “Experiment Settings,” the relationship between accuracy and number of firing neurons in the hidden layer (*Nf*_*hidden*_) is studied with the variation of network sparsity (Figure 5A), number of black pixels in the input images (Figure 5B), and the initial weights (Figure 5C), respectively. The sparsity study suggests that, when the sparsity is higher, *Nf*_*hidden*_ is larger. The best accuracy is achieved at sparsity 20%, where the corresponding *Nf*_*hidden*_ is the closest to 50% among the tested sparsity and the corresponding *f* values are close to one. The best accuracy is achieved at *Nin*_*b*_ = 20 and initial weight distribution *N* (0.0004, 0.0004), where the corresponding *Nf*_*hidden*_ value is the closest to 50% among the tested parameter ranges. These observations agree with the hypotheses. Figure 5D) shows the accuracy result with different combinations of the filter size, stride, and padding. The best result is given by filter size = 2, stride = 2, and padding = 0. This set of pre-processing parameters are used in this paper. Figure 5E shows that passing the gradient across a neuron only when *x*_*n*_ falls in a smaller range can help improve the accuracy. However, for living neural networks, only the upper bound constraint of the gradient could be implemented with the “cut-off” mechanism. Therefore, (0, 0.0075) is used for the following experiments in this paper. The result of hyper-parameter tuning is listed in Table 4, in the network and algorithm optimization part. With all the optimization methods, a 99.5% accuracy could be achieved for the 1000 MNIST dataset.

### Neural, Synaptic, and Network Variation Study

**FIGURE 6:**
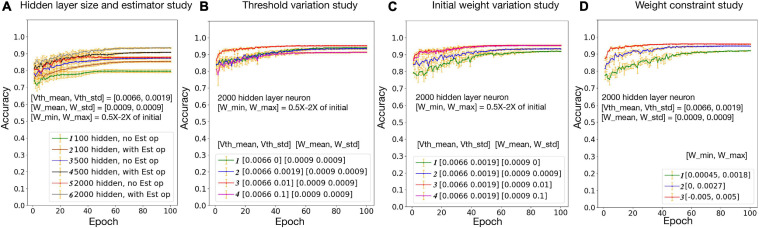
Neural, synaptic, and network variation study. Vth mean/Vth std: mean/standard deviation for biological layer threshold distribution; W mean/W std: mean/standard deviation for biological layer initial weight distribution; W min/W max: minimum/maximum weight constraint for biological layer.

The hybrid bio-silicon neural network learning accuracy for the full MNIST dataset is reported in Figure 6A after adding all optimizations discussed above. For 100, 500, and 2,000 hidden layer neurons, the average testing accuracy is 85.3, 90.8, and 93.4%, respectively. For the 2,000 hidden layer neuron case, a 6% accuracy gap is observed between training and testing accuracy. When increasing the hidden layer neuron number and adding the dropout technique to alleviate the overfitting issue, a 96% testing accuracy is achieved, which is a reasonable accuracy with biological constraints.

Effects of threshold and weight variations are evaluated separately in Figures 6B,C, respectively. Within the tested range, with the increase of the threshold variation, network accuracy slightly increases first and then starts to decrease. This is because a larger threshold variation brings too much noise to the network, which goes beyond the ability of the algorithm. With the increase of the synaptic weight variation, the network accuracy increases first then saturates. This is because the weights are trainable, and a larger initial weight variation enlarged the synapse changing space, since the weights are constrained to 0.5 × −2× of the initial weights. In Figure 6D, network accuracy is studied with the weight constraint. Synapse weights are initialized randomly but are constrained in a fixed range. Results suggest that, the larger the weight range, the higher the accuracy. Overall, a relatively good accuracy could be achieved with a realistic threshold, synapse weight, and weight constraints.

### Accuracy Comparison Between Biophysical and Computational Models After Optimization

**FIGURE 7:**
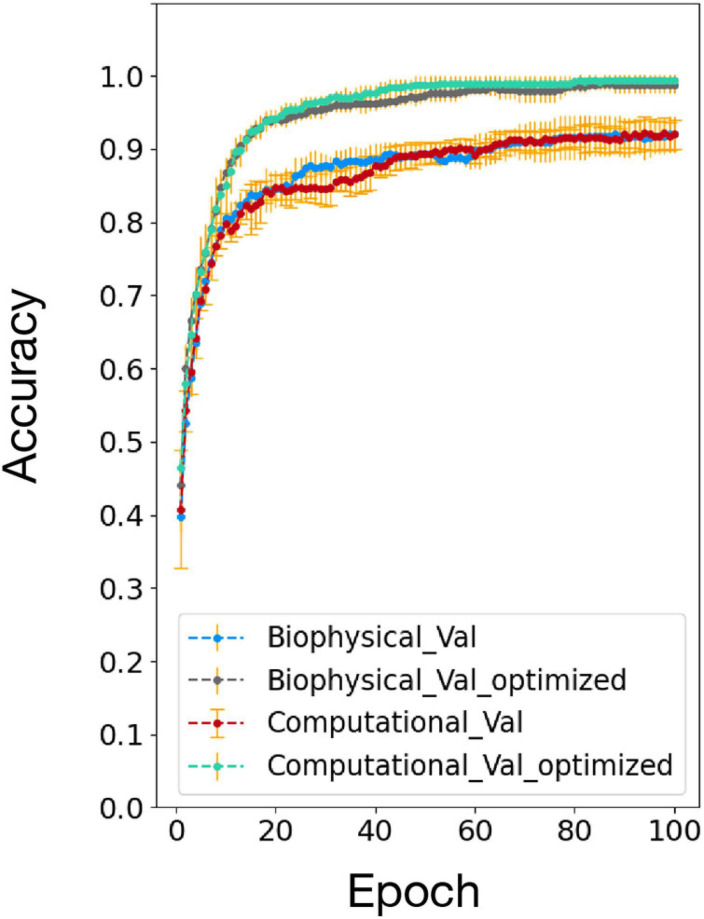
Accuracy comparison between biophysical and computational models after addition of adaptive pre-processing optimization (Biophysical Val optimized and Computational Val optimized).

The accuracy comparison result between the biophysical and computational model before and after the network and algorithm optimization is shown in Figure 7. After optimization, the testing accuracy and converge speed on the 100 MNIST dataset improved for both models. The accuracy also matched well between the biophysical and computational model after optimization. This verified the effectiveness of the optimization method on the biophysical model.

## Discussion

In this paper, a hybrid bio-silicon neural network is proposed and studied using both biophysical and computational models. Random network connections, as well as realistic threshold and synapse weight variations are considered. With proposed optimization methods, a 96% accuracy is achieved in simulation using a living neural network-fitted computational model. Simulation suggests that biologically plausible inference is not the major reason for a poorly performed bio-silicon computer; hence, living neurons could be used to design a learning machine.

This paper focuses on testing the influence of realistic biological properties on the inference process. Thus, the training approach is set to be the same as the conventional backpropagation algorithm to ensure a fair comparison between LNN and ANN accuracy. How to update the weights in a biologically plausible manner and ensure training efficiency is beyond the scope of this paper and will be carried out in our future work. Potential solutions could be updating weights through a supervised spike time-dependent plasticity (STDP) algorithm as shown in Zeng et al. (2018), or assigning blame by multiplying errors by random synaptic weights (Lillicrap et al., 2016). The functional equivalence between the NEURON-based biophysical model and the TensorFlow-based computation model are validated with a relatively small dataset of 100 MNIST images, because training the NEURON model is very time-consuming and the full MNIST simulation cannot be finished within a reasonable amount of time. Only the adaptive pre-processing method is applied to validate the computational optimization on the biophysical model. For a living neural network, the network topology cannot be easily controlled. Pre-processing guarantees a good accuracy with any measured network sparsity and it is the most effective optimization approach we found through the study shown in Figure 5. With the addition of adaptive pre-processing, the network accuracy on a small dataset of 100 MNIST images reaches 100%, therefore, other optimization approaches are not applied for biophysical model validation. This work focuses on the excitatory network, which has been studied extensively in the literature and can be experimentally implemented via inhibition of GABA*_*A*_* receptors (Goel and Buonomano, 2013; Odawara et al., 2016) as introduced in section “Algorithm.” With inhibitory synapse, the network activity will be more sparse and new learning features may emerge. The influence of the variability of inhibitory synapses on hybrid bio-silicon network performance is important and will be explored on the biological platform in our future work.

## Data Availability Statement

Publicly available datasets were analyzed in this study. This data can be found here: http://yann.lecun.com/exdb/mnist/.

## Ethics Statement

The animal study was reviewed and approved by Animal Care and Use Committee (IACUC) at Lehigh University.

## Author Contributions

YZ and XG developed the main concepts. YZ implemented the major part of the biophysical and computational models, performed the simulation, and drafted the manuscript. ZF and YB developed the biological platform. ZF performed the biological experiments and provided related data. WZ implemented the Chr2 channel in the biophysical model and helped in parameter tuning and adaptive pre-processing. MX helped in parameter tuning and adaptive learning rate. VP helped in converting biophysical variations into the computational model. All the other authors assisted in developing the concepts and writing the manuscript.

## Conflict of Interest

The authors declare that the research was conducted in the absence of any commercial or financial relationships that could be construed as a potential conflict of interest.
